# Targeting the DNA replication checkpoint by pharmacologic inhibition of Chk1 kinase: a strategy to sensitize APC mutant colon cancer cells to 5-fluorouracil chemotherapy

**DOI:** 10.18632/oncotarget.2475

**Published:** 2014-09-16

**Authors:** Estefania Martino-Echarri, Beric R. Henderson, Mariana G. Brocardo

**Affiliations:** ^1^ Centre for Cancer Research, University of Sydney, Westmead Millennium Institute at Westmead Hospital, Westmead, New South Wales, Australia

**Keywords:** 5-fluorouracil, APC, DNA replication checkpoint, Chk1 inhibitors, colon cancer

## Abstract

5-fluorouracil (5-FU) is the first line component used in colorectal cancer (CRC) therapy however even in combination with other chemotherapeutic drugs recurrence is common. Mutations of the adenomatous polyposis coli (APC) gene are considered as the initiating step of transformation in familial and sporadic CRCs. We have previously shown that APC regulates the cellular response to DNA replication stress and recently hypothesized that APC mutations might therefore influence 5-FU resistance. To test this, we compared CRC cell lines and show that those expressing truncated APC exhibit a limited response to 5-FU and arrest in G1/S-phase without undergoing lethal damage, unlike cells expressing wild-type APC. In SW480 APC-mutant CRC cells, 5-FU-dependent apoptosis was restored after transient expression of full length APC, indicating a direct link between APC and drug response. Furthermore, we could increase sensitivity of APC truncated cells to 5-FU by inactivating the Chk1 kinase using drug treatment or siRNA-mediated knockdown. Our findings identify mutant APC as a potential tumor biomarker of resistance to 5-FU, and importantly we show that APC-mutant CRC cells can be made more sensitive to 5-FU by use of Chk1 inhibitors.

## INTRODUCTION

Colorectal cancer (CRC) represents the third most lethal cancer in the world and affects both men and women. CRC results from complex interactions between inherited susceptibility and environmental or lifestyle factors [1, 2]. CRC develops through a sequential process often triggered in the initial step by mutations of the adenomatous polyposis coli (APC) tumor suppressor gene [3-5]. Single allele germ-line APC mutations are early events that lead to familial adenomatous polyposis, a relatively benign polyp syndrome, whereas mutation of both APC alleles predisposes to high risk of CRC [6]. CRC is one of the few cancers in which the initiation of tumor formation can be attributed to mutations of a single gene. Most APC mutations induce premature translation termination resulting in shortened N-terminal APC fragments that lack C-terminal sequences. Such truncated APC peptides display altered cellular localization, protein interactions and function. APC regulates normal function of the Wnt/beta-catenin signaling pathway [4, 7, 8] and also distinct from this pathway, functions as a mediator of apoptosis, cell cycle transition and the DNA damage/replication stress response and repair which influences its tumor suppressor activity [7-9]. We have previously reported that full-length APC contributes to the cellular response to DNA replication stress by stabilizing the association of replication protein A (RPA) complexes at stalled DNA replication forks after hydroxyurea treatment [10].

5-fluorouracil (5-FU) is a pyrimidine analogue that induces a DNA replication stress response in cells through its ability to inhibit thymidylate synthase. 5-FU is a baseline component of many first-line cancer chemotherapy regimens and even when it is used in combination with other chemotherapeutic agents, recurrence is common in CRC, especially in patients with metastasis due to the development of drug resistance [1, 11]. Therefore there is an intense interest in identifying strategies to enhance the initial response and/or to neutralize the emergence of resistance to 5-FU. A possible approach to increase 5-FU sensitivity is the pharmacological inhibition of DNA replication checkpoint signaling. Checkpoint kinase 1 (Chk1) is a key DNA damage checkpoint regulator activated by phosphorylation in two residues (Ser^317^ and Ser^345^) via ataxia telangiectasia and *Rad3*-related (ATR) kinase in response to single-strand DNA (ssDNA) produced either as intermediate forms of DNA damage or during stalled replication when dNTP levels are disrupted [12]. Numerous Chk1 small molecule inhibitors have been developed to enhance the activity of DNA-damaging agents but limited information exists about their role in the replication checkpoint induced by 5-FU in CRC cells [13-15].

In this work we report that CRC cell lines carrying APC mutations exhibit a reduced sensitivity to 5-FU. Cell apoptosis was restored by reconstituting the expression of full-length APC, implicating mutant APC as a determinant of cancer cell resistance to 5-FU. Moreover, our analysis of the response of APC-mutant cells to 5-FU revealed that (1) sensitivity to this drug was enhanced by inhibition of Chk1 kinase and (2) mutant APC at least partly contributes to the cell response to combined 5-FU and Chk1 inhibitor treatment by causing an increase in DNA replication fork stalling and/or collapse to trigger an apoptotic response.

## RESULTS

### Colon cancer cells exhibit differential sensitivity to 5-FU treatment depending on APC status

**Figure 1:**
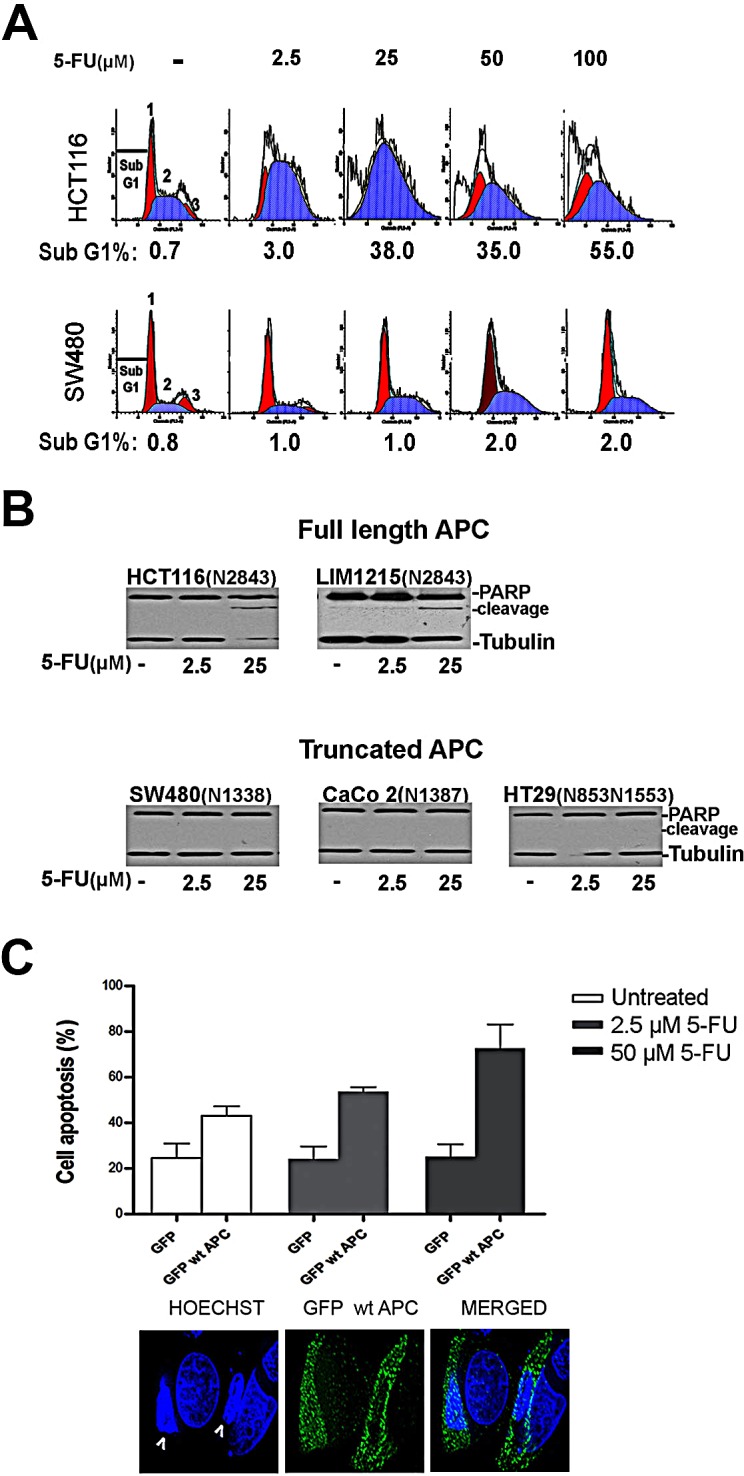
CRC cells are differentially sensitive to 5-FU treatment A, SW480 or HCT116 cells treated with increasing concentrations of 5-FU were stained with propidium iodide and analyzed by flow cytometry. G1, S and G_2_/M phase are indicated by 1, 2, 3 respectively. The experiment is representative of three. B, PARP detection by Western blot in colon cancer cell lines expressing full-length APC or truncated APC as indicated in parenthesis. C, Transient expression of GFP or GFP wild type APC (GFP wt APC) into SW480 cells treated with 2.5 μM or 50 μM of 5-FU for 36 h. At least 100 cells were counted. Positive transfected cells stained by Hoechst were scored for nuclear condensation. Proportion of apoptotic nuclei was represented in the bar graph. Representative figure shows SW480 cells expressing GFP wt APC and counterstained with Hoechst for nuclear visualization. Apoptotic cells are indicated with arrows.

To compare the effect of 5-FU on cell death and cell cycle arrest, we used flow cytometry analysis to detect changes in cell cycle profiles in two well characterized CRC cell lines: SW480 (express mutant APC) and HCT116 (express wild-type APC) cells. Following exposure to different concentrations of 5-FU for 36 h, substantial cell death (>35 % of cells) was observed in HCT116 cells using as little as 25 μM 5-FU whereas SW480 cells were resistant to the effects of 5-FU at all doses up to 100 μM (see sub-G1 peak in Fig. 1A). We observed that HCT116 cells accumulated in S phase after drug treatment while SW480 cells remained arrested at the G1/S boundary without undergoing lethal damage (Fig. 1A and Supplementary Fig. S1). We further evaluated the expression of PARP-cleavage by immunoblot analysis to confirm cell death by apoptosis in a panel of colon cancer cell lines. PARP-cleavage was clearly detectable by 5-FU treatment in cell lines expressing full-length APC (HCT116, LIM1215) but not in APC mutant cells (SW480, CaCo 2, HT29) (Fig. 1B).

To compare the impact of APC with that of p53 expression, since p53 is also mutated in several of the CRC lines analyzed, we knocked down wild type APC by siRNA in HCT116 cell lines wild-type or null for p53 and observed that APC depletion reversed the 5-FU-dependent induction of PARP cleavage in parental HCT116 cells similar to that observed in p53 null cells (Supplementary Fig. S2A). Thus, loss of full length APC can cause resistance to 5-FU. In order to determine if this was more than just a correlation, we examined whether the expression of wild-type APC in SW480 cells restored sensitivity to 5-FU. Cell death was determined on a cell-by-cell basis by Hoechst staining of nuclei to detect apoptotic DNA condensation. Increased SW480 cell apoptosis with 36 h 5-FU treatment was detected in the GFP wild type APC expressing cells compared to GFP controls (Fig. 1C and Supplementary Fig. S2B).

Moreover, to provide further evidence that the decrease in 5-FU sensitivity is caused by APC mutations, we induced the expression of Myc-tagged APC mutant (Myc-APCN1309) with tetracycline in a stable-transfected HEK293 cell line. This non-tumor epithelial cell line was sensitive to 25 μM 5-FU treatment and the expression of Myc-APCN1309 was observed to reverse the PARP-cleavage induced by 5-FU (Supplementary Fig. S3). This further indicates that expression of the mutant truncated form of APC can confer some resistance to 5-FU treatment. These results suggest first that CRC cells are differentially sensitive to 5-FU, and second that CRC cell lines carrying APC mutations are particularly resistant to 5-FU treatment.

### Chk1/2 inhibition sensitizes SW480 APC mutant CRC cells to 5-FU

**Figure 2:**
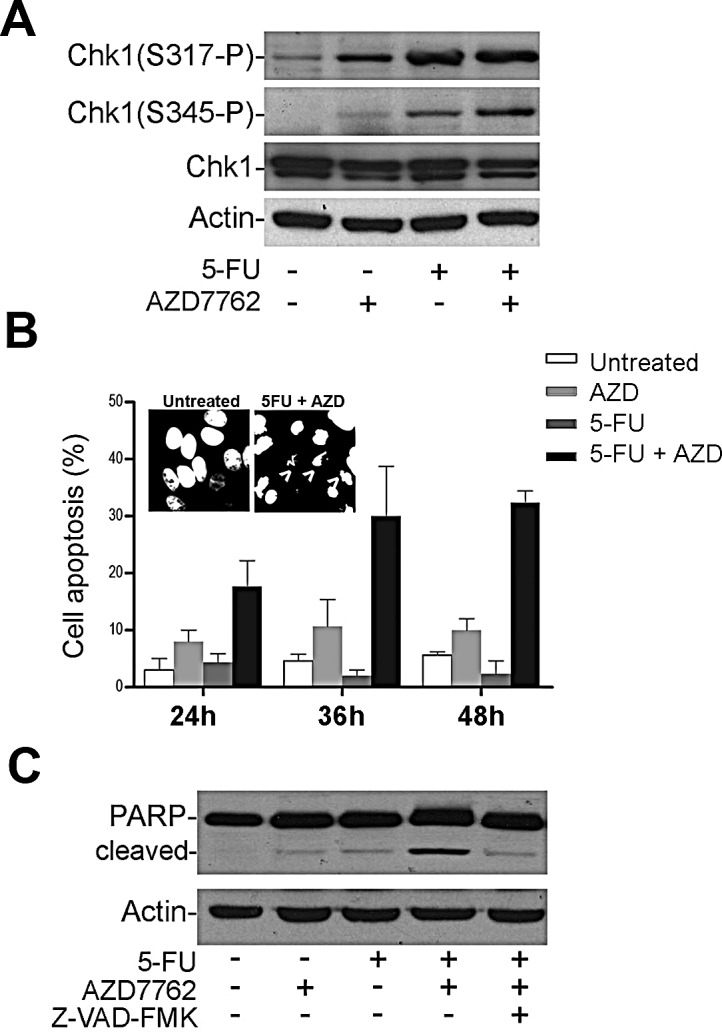
The Chk1/2 inhibitor AZD7762 sensitizes SW480 colon cancer cells to 5-FU A, SW480 cells were treated with 50 μM of 5-FU with or without 100 nM of AZD7762 (AZD). Cell lysates were immunoblotted to detect Chk1 phosphorylation. B, SW480 treated with 50 μM 5-FU in presence or absence of 100 nM of AZD7762 at different time points. Cells with condensed nuclei showed by Hoechst staining were considered apoptotic and were represented in the bar graph. Arrows indicated apoptotic cells. At least 200 cells were scored per sample from three different experiments. C, SW480 cells were treated similar to A and B in presence or absence of 20 μM of the pan-caspase inhibitor Z-VAD-FMK (Z-VAD) to detect PARP cleavage product by Western blot analysis.

Next, we investigated whether the sensitivity to 5-FU in APC mutant CRC cells could be increased by further disruption of the DNA replication checkpoint. When the DNA is damaged during S phase, Chk1 plays a prominent role in restraining initiation of DNA replication. Both APC and Chk1 are involved in the cellular response to DNA replication stress through their association with the 32 kDa subunit of the replication protein A (RPA) [16] [10, 12]. Thus, we tested the pharmacological inhibition of Chk1 as a potential approach to trigger an apoptotic response in mutant APC CRC cells that were arrested with 5-FU. We first confirmed that 5-FU induced ATR-mediated phosphorylation of Chk1 at Ser^317^ and Ser^345^ in SW480 cells (Fig. 2A) and showed that the level of phosphorylation observed was higher than that seen in HCT116 cells (Supplementary Fig. S4), suggesting that APC-mutant SW480 cells may be more reliant on the Chk1 checkpoint pathway. Next, we investigated whether AZD7762, an ATP competitive Chk1/2 inhibitor, was able to sensitize SW480 cells to 5-FU. After addition of 100 nM AZD7762 alone, the Chk1 phosphorylation level (Fig. 2A) and level of apoptosis (Fig. 2B) was only weakly enhanced, however when used in combination with 5-FU it induced a striking~15-fold increase of apoptotic nuclei compared to cells treated with 5-FU alone (Fig. 2B). The combined action of AZD7762 and 5-FU also stimulated PARP cleavage in SW480 cells (Fig. 2C). The pan-caspase inhibitor Z-VAD-FMK reduced PARP cleavage and the number of apoptotic nuclei, confirming that this is a caspase-dependent apoptotic process (Fig. 2C and data not shown). The results suggest that 5-FU induces Chk1 phosphorylation, and that Chk1/2 inhibition by AZD7762 treatment sensitizes SW480 APC mutant cells to 5-FU-mediated apoptosis.

### Selective Chk1 inhibition sensitizes CRC cells to 5-FU induced apoptosis

**Figure 3:**
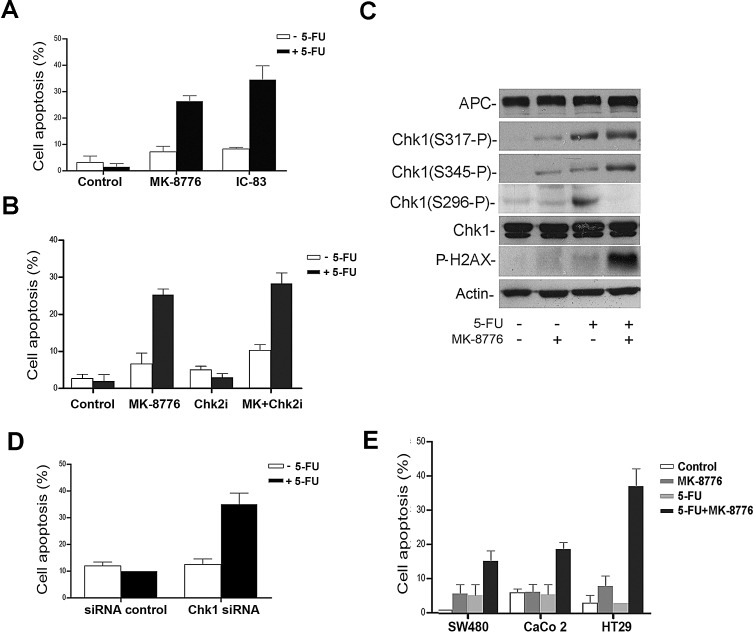
The selective Chk1 inhibition sensitizes CRC cells to 5-FU induced apoptosis A, SW480 cells treated with 5-FU in presence of 500 nM of MK-8776 or 500 nM of IC-83 Chk1 inhibitors. The condensed nuclei stained with Hoechst were considered apoptotic. At least 400 cells were counted per sample in three separate experiments and represented in the bar graph. B, 5-FU-arrested SW480 cells were treated with 500 nM of MK-8776 or 10 uM of Chk2 inhibitor (Chk2i). Cells were scored for nuclear condensation. Proportion of apoptotic nuclei was represented in the bar graph. C, Detection of different forms of Chk1 phosphorylation: Ser296 (S296-P), Ser317 (S317-P) and Ser345 (S345-P) and gamma-H2AX by immunoblotting of SW480 cells treated with 500 nM of MK-8776 and 50 μM of 5-FU. D, siRNA control or Chk1 siRNA were transfected in SW480 cells and treated with 50 μM of 5-FU. Cells were analyzed and nuclear condensation was detected by immunofluorescence. Proportion of apoptotic nuclei was represented in the bar graph. E, Apoptotic nuclei were scored in cells treated with a sub-optimal 5-FU concentration of 2.5 μM in combination with MK-8776. At least 200 cells were scored per sample from three different experiments. Proportion of apoptotic nuclei was represented in the bar graph.

Since AZD7762 inhibits both Chk1 and Chk2 kinases, we tested more selective Chk1 inhibitors. The treatment of SW480 cells with the Chk1 inhibitor MK-8776 (SCH900776) was also found to sensitize cells to 5-FU, and the combined action of the two drugs increased the number of apoptotic cells and PARP cleavage product with an impact similar to that of AZD7762 (Fig. 3A and Supplementary Fig. S5). In parallel experiments another Chk1 inhibitor, IC-83 (LY2603618), elicited similar results (Fig. 3A). In contrast, the selective Chk2 inhibitor (Chk2 inhibitor II) was not able to sensitize SW480 cells to 5-FU (Fig. 3B) despite confirmation of its activity by reduction of p53 Ser20 phosphorylation a known substrate of Chk2 (Supplementary Fig. S6). The efficacy of the Chk1 inhibitor MK-8776 was confirmed by immunoblotting, and the drug was shown to inhibit Chk1 autophosphorylation (Ser^296^) that is required for reducing Chk1 activity, but had little effect on ATR-mediated Chk1 phosphorylation (Fig. 3C). We also investigated the phosphorylation of histone gamma-H2AX, a downstream signal for checkpoint abrogation and marker for both single strand and double strand breaks. 5-FU and MK-8776 alone each induced only a slight increase in gamma-H2AX levels in control cells, whereas the combination of both drugs caused a dramatic increase in gamma-H2AX signal consistent with an enhanced cell death induced by 5-FU (Fig. 3C). To ensure that the effects of Chk1 inhibitor observed were a direct consequence of Chk1, the expression of Chk1 was down-regulated by using short interference RNA (siRNAs) and evaluated by immunoblotting. Neither 5-FU nor Chk1 siRNA alone induced apoptosis. In contrast, the combination of 5-FU and Chk1 siRNA increased the number of condensed nuclei to levels similar to that observed after Chk1 inhibition by drug treatment (Fig. 3D and Supplementary Fig. S7).

Finally, we tested the specific Chk1 inhibitor combined with a much lower 5-FU concentration of 2.5 μM, considered within the clinical therapeutic range in CRC [17]. Even at this concentration, a significant sensitization to 5-FU was observed in all APC-mutant CRC cell lines tested, particularly in HT29 cells (Fig. 3E). Notably, this low dose 5-FU combination did not adversely affect normal epithelial HEK293 cells or HDF1314 fibroblasts, providing an indicator of a therapeutic window (Supplementary Fig. S8A). HCT116 cells expressing wild type APC displayed a more modest response to the combination drug treatment (Supplementary Fig. S8B). This result demonstrates that the efficacy of 5-FU may be enhanced by Chk1 inhibitors in CRC cell lines expressing APC truncations, although it should be noted that the 5-FU sensitivity of cells after over expression of wild type APC was not further enhanced by inhibition of Chk 1 (Supplementary Fig. S8C).

### Chk1 inhibition allows 5-FU-treated SW480 cells to stimulate the collapse of stalled replication forks to trigger an apoptotic response

**Figure 4:**
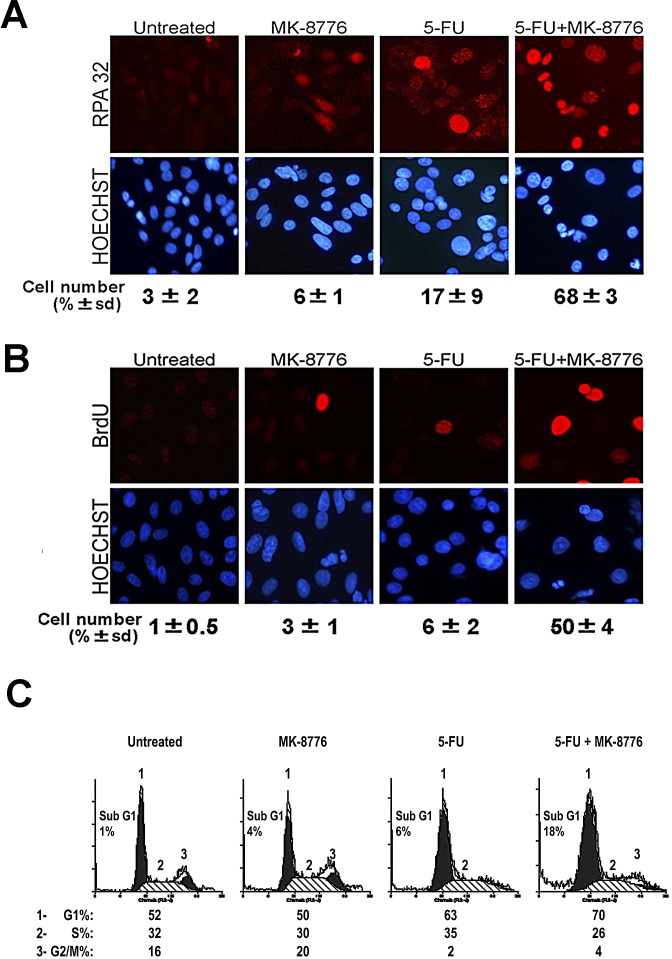
Chk1 inhibition allows 5-FU-treated SW480 cells to stimulate the collapse of stalled replication forks to trigger an apoptotic response A, Representative images of RPA foci obtained by immunostaining of SW480 cells treated with 5-FU in presence or absence of MK-8776 Chk1 inhibitor. More than 100 cells were counted for each condition. The scoring showed underneath represents the average of two experiments. B, Cells were similarly treated as in panel A and stained for BrdU and nuclei C, Cell cycle profile of SW480 cells treated with 5-FU, MK-8776 or a combination of both drugs.

In further experiments we examined the underlying process involved in the sensitization to 5-FU by Chk1 inhibitors. Chk1 activation is triggered by formation of RPA-ssDNA regions which are considered early events in the response to DNA replication stress [18]. Therefore, we used fluorescence microscopy to detect RPA foci and BrdU incorporation as markers of replication stress and DNA synthesis, respectively. Indeed, as expected (Fig. 4A), 5-FU treatment alone caused an increase in nuclear RPA foci (detected with antibody against the middle subunit RPA32), and this was increased dramatically in the presence of the Chk1 inhibitor. The accumulation of RPA32 foci correlated with an increase in BrdU incorporation (Fig. 4B). This data suggest that while the unregulated origin firing may lead to an increase in DNA synthesis it may also be possible that the drug-induced stalling and collapse of the replication forks generate more ssDNA to which RPA is able to bind. Moreover, the prolonged increase in ssDNA may provide more substrate for repair synthesis, leading to greater BrdU incorporation. By flow cytometry analysis, the proposed impairment of the replication checkpoint by 5-FU combined with MK-8776 correlated with a reduction in the percentage of cells in S phase and an increase of those in Sub-G1 (Fig 4C). Similar results were observed using AZD7762 (Supplementary Fig. S9). These results are consistent with the notion that while 5-FU arrests SW480 cells in early S-phase at DNA replication, the addition of the Chk1 inhibitor appears to increase the concentration of ssDNA consistent with prolonged stalling/collapse of replication forks, and thus the cells exposed to 5-FU are unable to complete the cycle of DNA synthesis and subsequently undergo apoptosis.

### Mutant APC has a gain-of-function effect and contributes to optimal apoptotic response to 5-FU after Chk1 inhibition in SW480 cells

**Figure 5:**
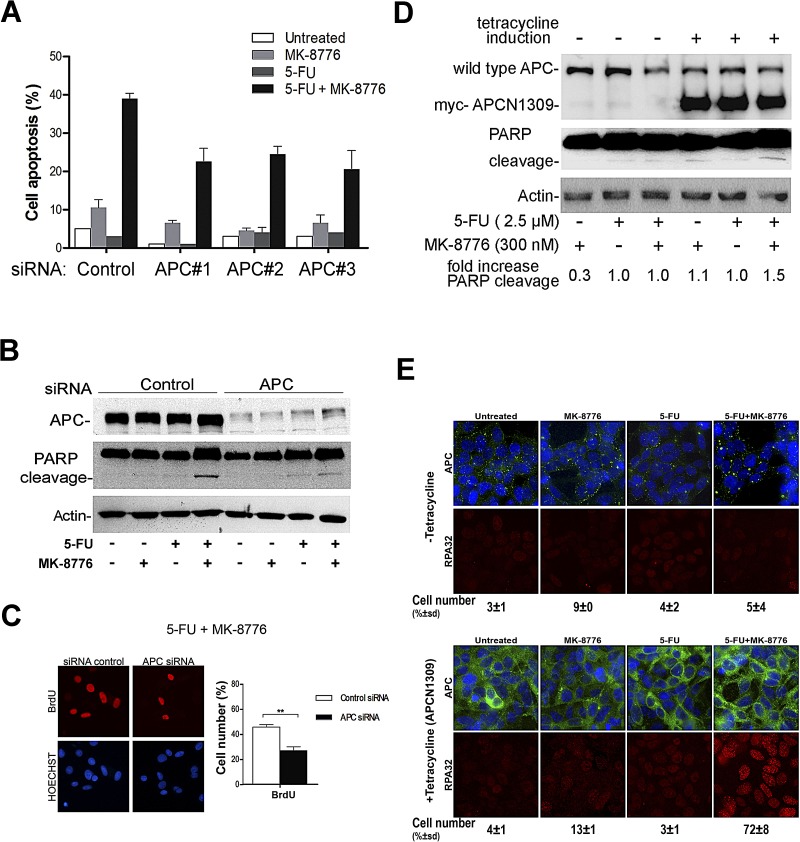
Mutant APC has a gain-of-function effect and contributes to optimal apoptotic response to 5-FU after Chk1 inhibition in SW480 cells A, SW480 cells were transfected with siRNA control or different APC siRNA (#1, #2 or #3). After 24 h, cells were treated with 50 μM of 5-FU in presence of 500 nM of MK-8776 and stained with Hoechst to detect apoptosis by nuclear condensation. Proportion of apoptotic nuclei was represented in the bar graph. B, SW480 cells were transfected with siRNA (control or APC#1) and treated with 500 nM of MK-8776, 50 μM of 5-FU or a combination of both drugs to detect PARP-cleavage by immunoblotting. C, siRNA transfection and drug treatments were carried out similar to explained in A. The cells were fixed and stained to detect BrdU. Bar graph shows the proportion of cells showing BrdU staining **, P < 0.001 (two-way ANOVA) D, inducible 293HEK cells stably expressing myc-tagged N1309, were induced with 2 μg/ml of tetracycline overnight and treated with 300 nM of MK-8776, 2.5 μM of 5-FU or a combination of both drugs to detect PARP-cleavage by Western blot analysis. E, inducible 293HEK were treated similar to described in 5D. Representative images of RPA foci (in red), APC (in green) and Hoechst (in blue) were obtained by immunostaining. For each condition 100 cells were count. The scoring showed underneath represents the average of two experiments.

We earlier showed that not only did loss of wild-type APC increase resistance of HCT116 CRC cells to the effects of 5-FU (Supplementary Fig. S2A), but that overexpression of mutant APC(1-1309) could increase 5-FU resistance in non-tumor HEK293 cells (Supplementary Fig. S3). However, the influence of mutant APC expression on the overcoming of 5-FU resistance after Chk1 inhibition was unclear. To address this, we first silenced mutant APC in SW480 cells using different siRNAs to control for off-target effects and compared the response to 5-FU drug alone. APC depletion was confirmed by Western blot analysis (Supplementary Fig. S10). As shown in Fig. 5A, down-regulation of mutant APC did not enhance the response to 5-FU alone, indicating that continued expression of APC mutants is not always required to maintain drug resistance. Conversely, the depletion of mutant APC decreased the level of apoptosis mediated by 5-FU in presence of MK-8776 in SW480 cells, reducing it from ~40% to ~25% (Fig. 5A). This indicates that continued expression of mutant APC partially contributes to overcoming 5-FU resistance on addition of the Chk1 inhibitor. Under similar conditions a decrease of PARP-cleavage was detected upon knockdown of mutant APC (Fig. 5B), and this correlated with a reduction in BrdU incorporation (Fig. 5C).

Next, we tested the impact of overexpressing mutant APC in normal cells. Myc-tagged APC(N1309) was induced by tetracycline in a stable HEK293 cell line. The expression of mutant APC had little effect on HEK293 cells treated with either 5-FU or MK-8776 alone, however in cells treated with the combination of 5-FU and Chk1 inhibitor the induction of mutant APC induced both PARP-cleavage and RPA foci formation (Fig. 5D and E). This response was only observed after induction of the APCN1309 mutant and resembled the effects of the drugs seen in SW480 cells (Fig's. 3 and 4). When considered together, the data suggest that continued expression of mutant APC contributes to enhanced sensitivity of SW480 cells to combined 5-FU and Chk1 inhibitor treatment.

## DISCUSSION

The effectiveness of 5-FU has improved the overall survival rates of colon cancer patients however there are serious limitations such as widespread tumor resistance and toxicity, resulting in a narrow therapeutic window [1, 2]. Therefore, the predicted efficacy of 5-FU using reliable biomarkers and more effective strategies is needed. Here we have identified one such biomarker and provide *in vitro* evidence that the presence of APC mutations prevents 5-FU sensitivity. Indeed, we show that the loss of wild type APC and the expression of mutant truncated APC both contribute to 5-FU resistance, while reinstating expression of full-length APC restores 5-FU induced apoptosis. Thus in future, the restoration of APC through techniques such as gene therapy or the induction of read-through stop codons may be of therapeutic benefit for APC-mutant cancers [19]. In this work, we report that targeting the DNA replication checkpoint followed by Chk1 inhibition overcomes 5-FU resistance in mutant APC cells and this has potentially far reaching clinical implications, as combination drug therapies might benefit those patients currently not responding to 5-FU.

Chk1 knock down by siRNA was previously shown to enhance cell death in HeLa and in CRC to arrest cell growth [20, 21]. However, this kinase has critical roles in a broad range of cellular processes therefore our findings indicate that the transient inhibition of Chk1 by small molecules may be preferable to the toxic effects caused by permanent Chk1 ablation. Chk1 inhibitors have previously been tested in a range of cancer cell lines and shown to varying extents to improve cellular sensitivity to different DNA damaging chemotherapeutic agents in some cases boosting sensitivity to agents such as hydroxyurea or gemcitabine but not to 5-FU in CRC [22-24]. Moreover, Guzi *et al*. reported that MK-8776 in combination with hydroxyurea did not lead to a dramatic increase of cell death in WS1 fibroblasts, suggesting that certain combinations of Chk1 inhibitors and DNA replication blockers may selectively target specific types of cancer cells [23], as further revealed in this study. Here, we showed for the first time that mutant APC is involved in the ATR-Chk1 signaling pathway by protecting cells from 5-FU cytotoxicity. We also found that in certain APC-mutant CRC cell lines the pharmacologic inhibition of Chk1 can magnify 5-FU efficacy and that the continued expression of truncated APC protein contributes to this process, driving cells to undergo apoptosis. The overcoming of 5-FU resistance was accompanied by increased expression of early markers of DNA replication stress events such as RPA32 and gamma-H2AX, suggesting that the replication checkpoint is rapidly compromised and can occur before cells enter in apoptosis. The increase in cell death that we observed was also achieved using therapeutically relevant concentrations of 5-FU, indicating that the potential side effects and costs often associated with CRC treatment could be minimized and controlled.

Chk2 is also activated by ATR/ATM kinases and shares some common substrates with Chk1. Although our findings excluded an apoptotic role for Chk2 inhibition, we do not rule out the possibility that Chk2 may be involved in other aspects different from checkpoint replication such as DNA repair [25, 26].

The mechanisms by which cellular apoptosis and DNA replication stress are potentiated or retarded by APC mutants remain unresolved. Several chemotherapeutic agents that inhibit DNA replication such as methylmethane sulfonate (MMS) and oxaliplatin also impair the DNA base excision repair (BER) system and promote apoptosis. It was shown that APC binds to polymerase beta and contributes to enhance the action of these drugs by inhibiting the BER system. However, the effectiveness of these treatments is impaired in APC-mutant cancers as a result of a functional BER capacity and should be considered when these drugs are administrated [27, 28]. A recent report suggests that the homologous recombination repair (HRR) process has multiple functions at stalled replication forks and can contribute to 5-FU tolerance in DT40 B lymphoma cells. Chk1 inhibition was found to exacerbate the effect of 5-FU by stimulating formation of H2AX foci and cytotoxicity, not only in wild type cells but also in HRR-deficient cells indicating that further understanding of how the ATR-Chk1 pathway impacts on 5-FU toxicity has implications for a range of cancers including those with APC mutations [29]. Future experiments will determine the overlap between mutant APC and Chk1 and whether APC regulates Chk1-dependent replication stress and repair.

We and others reported that a decrease of mutant APC levels achieved by siRNA impairs the regulation of DNA replication checkpoint components such as RPA32 and the proliferating cell nuclear antigen (PCNA) and also showed that APC associates with PCNA and RPA32 [10, 30]. A decrease in the number of DNA replication foci after Chk1 inhibition in APC depleted cells could indicate that mutant APC may promote an increase stalling/collapse of replication forks in the APC-mutant CRC cells, as suggested by increased ssDNA detected by RPA and BrdU assays. Such changes may stimulate apoptosis.

The effect of 5-FU on cell growth arrest and apoptosis was previously attributed to the ability of this drug to induce the expression and activity of the tumor suppressor p53 linked to later stages of tumorigenesis [31, 32]. In this study we compared the effects of depleting either wild-type APC or p53 in HCT116 CRC cells, and found that loss of either protein increased resistance to 5-FU treatment (Supplementary Fig. S2). Thus, although mutations in p53 are also frequent in colon cancer, the fact that Chk1 inhibition can influence cell apoptosis independent of p53 status suggests that a strategy combining Chk1 inhibitors with 5-FU may be a novel alternative to treat CRC with both APC and p53 mutations [23, 25, 33]. In conclusion, these data have identified mutant APC as a tumor biomarker that may predict poor responders to the common 5-FU treatment and we propose that the inhibition of Chk1 targeting selectively APC-mutant populations in colon cancer cells may provide a more effective strategy for chemosensitization to 5-FU.

## MATERIAL AND METHODS

### Cell lines and reagents

The human colorectal cancer lines HCT116, SW480, HT29, CaCo 2, LIM1215 were obtained from the American Type Culture Collection and culture according to their protocols. The cell lines were authenticated by STR analysis. Stable inducible HEK293 cell lines were created as described previously [34]. Cells were selected in 15 μg/ml blasticidin (Fluka) and 150 μg/ml hygromycin B (Invitrogen). Protein expression Myc-tagged-APC (N1309) was induced by the addition of 2 ng/ml tetracycline (Sigma).

5-fluorouracil (5-FU) (Sigma); AZD7762, MK-8776 (SCH900776) and IC-83 (LY2603618) (Pfizer); Chk2 Inhibitor II hydrate (Sigma); Caspase Inhibitor Z-VAD-FMK (R&D Systems). Primary antibodies for 1:1000 rabbit monoclonal anti-phospho-Chk1 (Ser345-P) (133D3) (# 2348, Cell Signaling), 1:1000 rabbit polyclonal anti-phospho-Chk1 (Ser317-P) (# 2344, Cell Signaling), 1:1000 rabbit polyclonal anti-phospho-Chk1 (Ser296-P) (# 2349, Cell Signaling), 1:1000 rabbit polyclonal anti Chk1 (#2345, Cell Signaling), 1:10000 rabbit polyclonal Chk1 (#A300-298A, Bethyl), 1:1000 rabbit polyclonal PARP (#9542, Cell Signaling), 1:2000 mouse monoclonal anti-β-actin (A5316, Sigma), 1:100 mouse monoclonal anti-APC (Ab1) (# OP44, Cabiochem), 1:500 rabbit polyclonal anti-APC (# H-290, Santa Cruz), 1:1000 rabbit polyclonal anti-phospho-p53 (Ser20-P) (#9287, Cell signaling), 1:100 mouse monoclonal anti-Bromo-deoxyuridine (BrdU) (#RPN202, Amersham) or 1:400 monoclonal anti-RPA32 (NA18; Calbiochem).

### Small interfering RNA transfection (siRNAs)

Cells were plated at 50 % of confluence in 25 cm^2^ and transfected using Lipofectamine^TM^ (Invitrogen) according to supplier instructions. After 6 h, medium was replaced and drug treatment was applied for 36 h. The siRNA were: Human Chk1 siRNA SMART pool or RNA duplexes (combination of 3 of them to avoid off-targets responses) (Dharmacon Technology); double-stranded-21-mer RNA oligonucleotides homologous to sequences in human APC and siRNA control (Qiagen) were: APC#1 5′-AACGAGCACAGCGAAGAATAG-3′ corresponding to nucleotides 691–714, APC#2 5′-AGGGGCAGCAACTGATGAAAA-3′ corresponding to nucleotides 5887–5896 and APC#3 (5′-AGCCGGGAAGGATCTGTATCA-3′ nucleotides 330-350). The sequence used as control was: 5′-AACGAGCAGTCGCTTCAATAG-3′.

### Plasmid transfection

The plasmid GFP wild type APC (APC amino acids 1–2843): was kindly supplied by Dr. Angela Barth. GFP wild type p53 plasmid (12091, Addgene) [35]. The cells were seeded at 75 % confluence and transfected with 1 μg/ml of DNA using FuGENE HD reagent as instructed by the supplier (Promega). After 6 h, the medium was replaced with the drug treatment for 36 h.

### Cell collection and processing

After treatment, floating and adherent cells were combined by centrifugation and the pellets were washed and suspend in PBS.

### -Cell cycle analysis

20 % of the above suspension was fixed in 85 % iced-cold ethanol, and stored at −20 °C for up 2 weeks for flow cytometry analysis. After centrifugation to remove the ethanol the pellets were suspended in PBS containing 50 μg/ml RNAase A (Worthington), 50 μg/ml propidium iodide (Sigma) and incubated at 37 °C for 1 h. Flow cytometry was carried out using FACSalibur (Becton Dickinson) and Modifit LT 3.1 and Cell Quest software. At least 7000 events were gated.

### -Western Blotting

80 % of the initial cell suspension was pellet and lysed with RIPA buffer. The protein suspension was centrifuged and the supernatants collected and protein quantified using a Bradford solution. Equal amount of protein were separated via SDS-PAGE and then transferred to nitrocellulose membranes. The membranes were blocked for 1 h in blocking buffer (1 x TBS, 3 % milk, 0.1 % Tween 20) and placed in primary antibody diluted in blocking buffer, overnight at 4 °C. The following day, membranes were washed three times in wash buffer (0.1 % Tween 20, 1 x TBS). Primary antibody was detected using horseradish peroxidase linked secondary antibodies and developed using enhanced chemiluminescence (ECL) detection reagents according to manufacturer instructions (Amersham).

### Immunofluorescence analysis

Cells were seeded on coverslips at 50 % confluence 24 h prior to the addition of drugs for 36 h. Before fixation BrdU pulse was applied and the cells were incubated for 1 h with cell proliferation labelling reagent Bromo-deoxyuridine (BrdU) (# RPN201, Amersham, GE Healthcare). In those assays designed for BrdU and RPA32 detection the cells were first treated for 5 min on ice with CSK buffer (10 mM HEPES/KOH, pH 7.4, 300 mM sucrose, 100 mM NaCl, 3 mM MgCl_2_, 0.5 % Triton X-100 supplemented with protease inhibitor cocktail) buffer to achieve better visualization of foci and then fixed with 3.7 % formaldehyde for 20 min. Antibody dilutions and washes after incubations were performed in PBS containing 1 % BSA. Cells were fixed and stained with Hoechst to detect the nuclear condensation. Coverslips were finally mounted in Vectashield mounting medium (H-1500; Vector Laboratories). A SPOT camera and SPOT Advanced software was used for general image capture. For the experiment showed in Figure 1C and Figure 5E, Olympus DeltaVision core deconvolution microscope with SoftworxResolve 3D software was used for advanced imaging analysis. Deconvolved cell images were compiled in Adobe Photoshop CS.

## SUPPLEMENTARY FIGURES


