# Supplementary material for: Targeting the DNA replication checkpoint by pharmacologic inhibition of Chk1 kinase: a strategy to sensitize APC mutant colon cancer cells to 5-fluorouracil chemotherapy
Source: Oncotarget. 2014 Sep 16;5(20):9889–900. doi: 10.18632/oncotarget.2475 (PMC4259445; doi:10.18632/oncotarget.2475)
Supplement: Supplementary file 1 [file oncotarget-05-9889-s001.pdf]

Targeting the DNA replication checkpoint by pharmacologic inhibition of Chk1 kinase: a strategy to sensitize APC mutant colon cancer cells to 5-fluorouracil chemotherapy

Supplementary Material

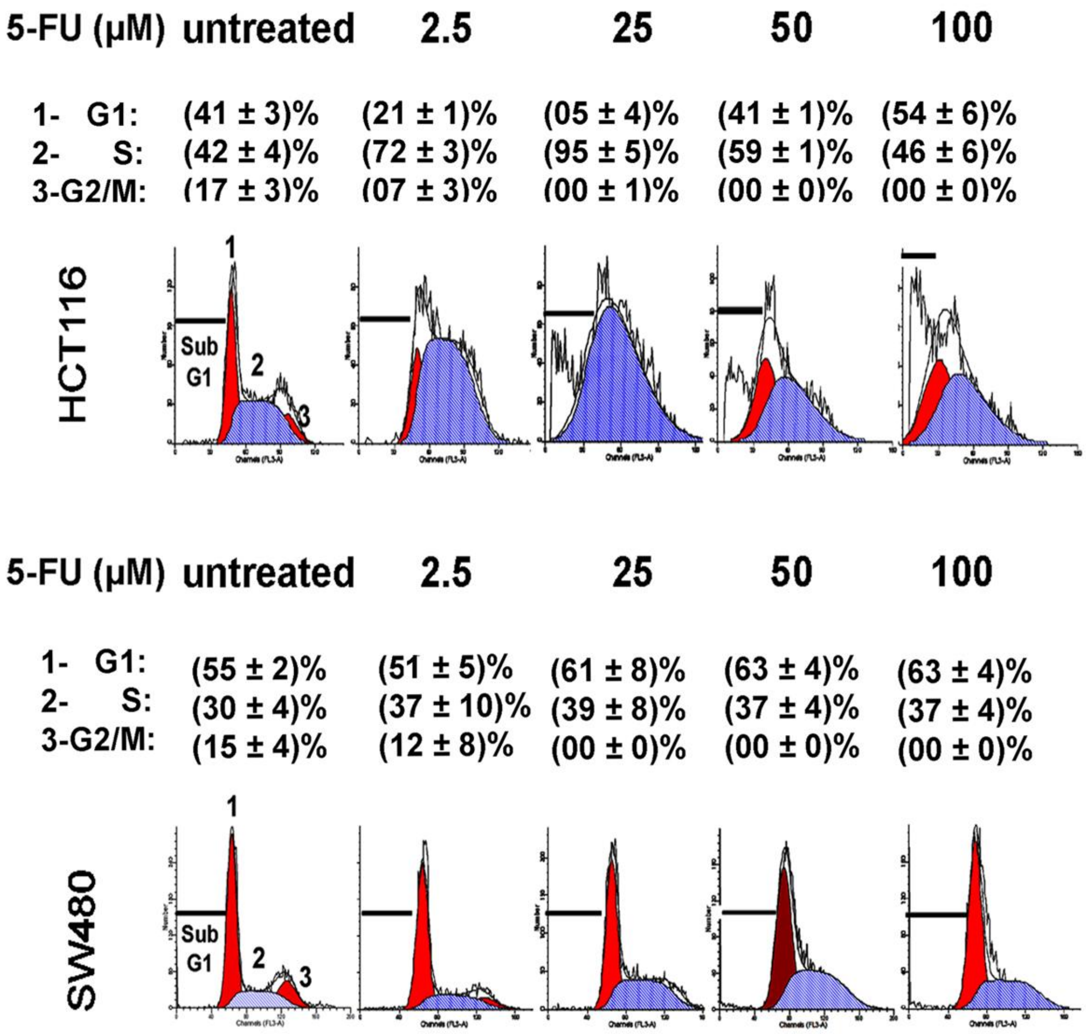

Supplementary Figure S1: SW480 and HCT116 cells treated with different concentrations of 5-FU. Cell cycle profile by flow cytometry is showed for both cell lines. The data represent the average of three experiments (mean ± sd).

A

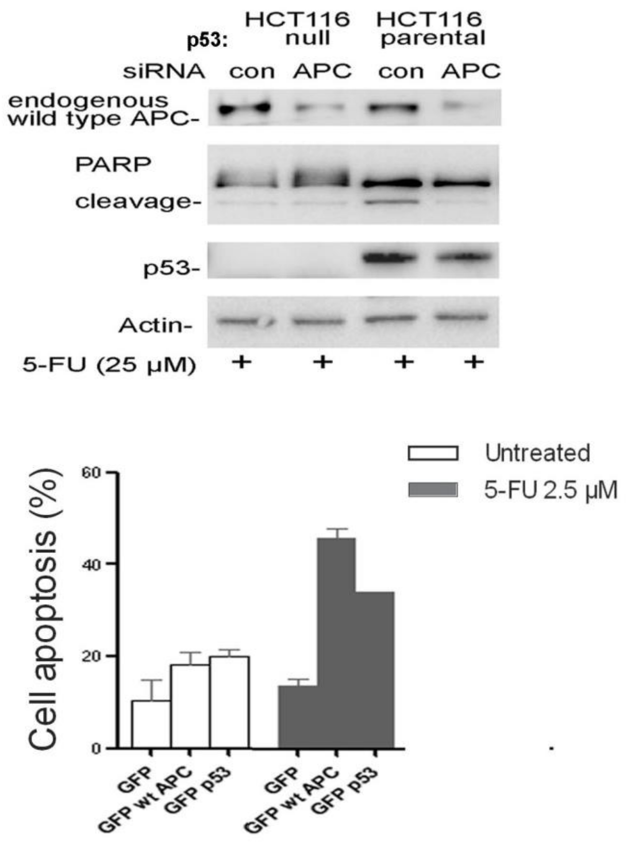

Supplementary Figure S2: A, HCT116 cells (p53 null or parental) were transfected with siRNA (control or APC). 24 h later the cells were treated with 25 µM of 5-FU for other 36 h to induce apoptosis. Down-regulation of APC, PARP-cleavage and p53 was detected by Western blot analysis. B, Transient expression of GFP, GFP wild type APC (GFP wt APC) and GFP wild type p53 (GFP p53) into SW480 cells treated with 2.5 µM for 36 h. At least 100 cells were counted. Positive transfected cells were stained by Hoechst scored to detect nuclear condensation. Proportion of apoptotic nuclei was represented in the bar graph.

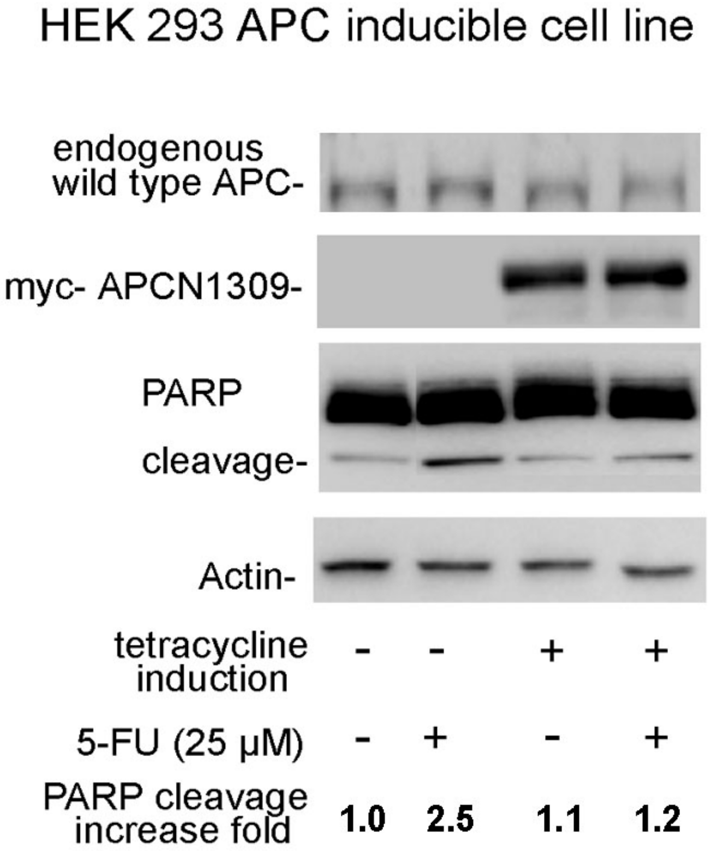

Supplementary Figure S3: Stable inducible HEK293 cells were induced with tetracycline to expressed Myc-tagged-APC (N1309) and treated 25 µM of 5-FU. Detection of APC and PARP-cleavage was carried out by Western blot analysis. The increase fold number represents the increase of PARP-cleavage in comparison to the control and normalized for actin.

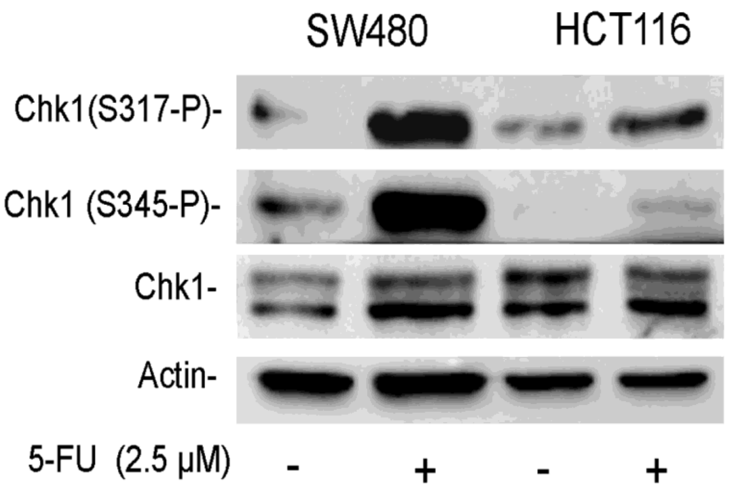

Supplementary Figure S4: Western blot detection of the phosphorylated forms of Chk1 at Ser^{317} and Ser^{345} in SW480 and HCT116 cells treated with 2.5 µM of 5-FU for 36 hours.

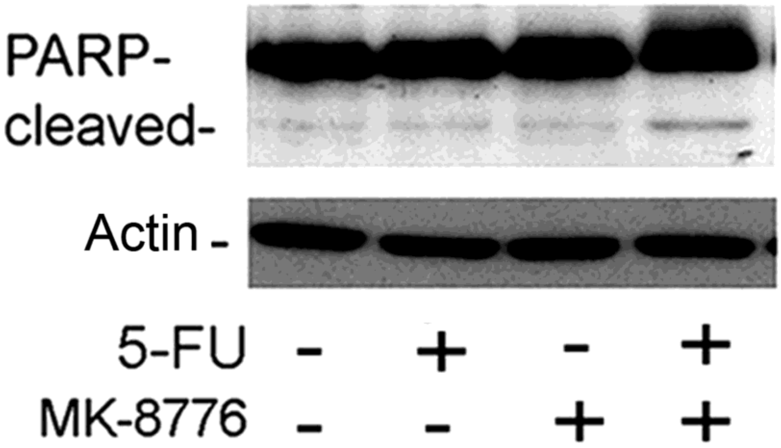

Supplementary Figure S5: Detection of PARP-cleavage by Western blot analysis in SW480 cells treated with 500 nM of MK-8776 and 50 µM of 5-FU.

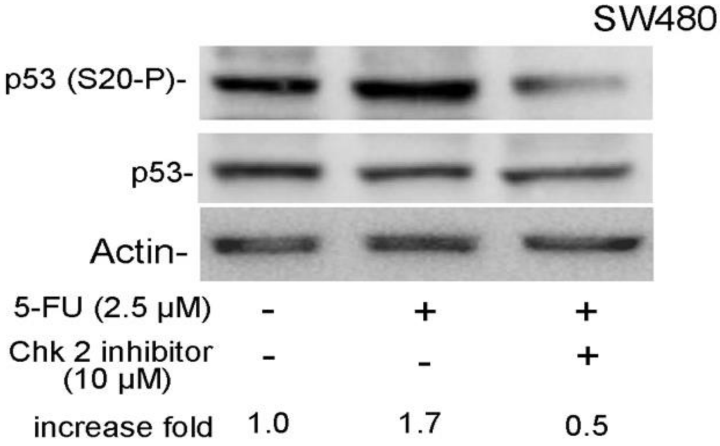

Supplementary Figure S6: SW480 cells were treated with 10 µM of Chk2 inhibitor drug in presence of 2.5 µM of 5-FU and phosphorylation of p53 at Ser^{20} was detected by Western blot. The increase fold number represents the increase of phosphorylation in comparison to the control and normalized for actin.

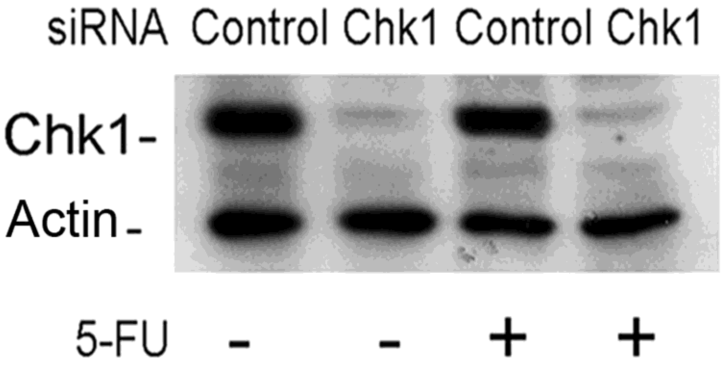

Supplementary Figure S7: SW480 cells were transfected with siRNA (control or Chk1) and treated with 50 µM of 5-FU. Chk1 was depleted using a smart pool composed by 3 different siRNAs. Chk1 depletion was detected by Western blot analysis.

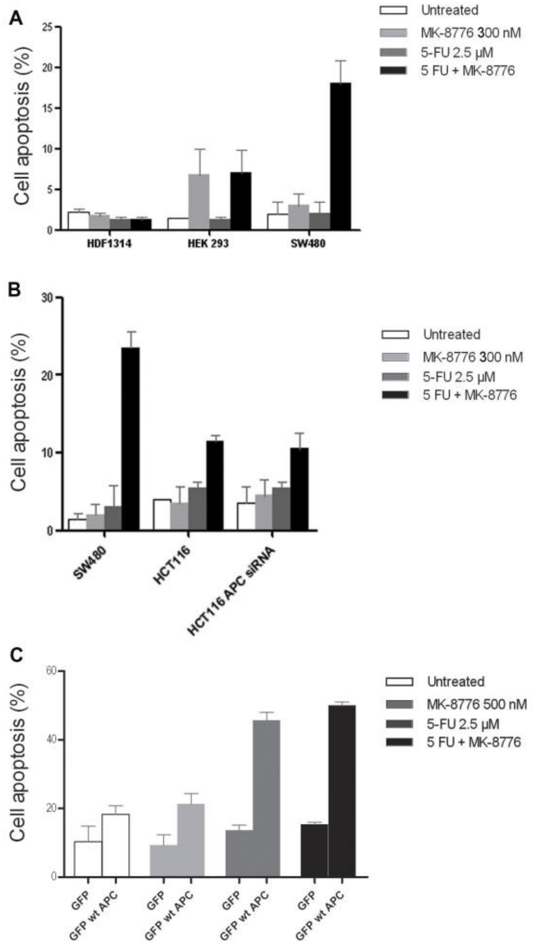

Supplementary Figure S8: A, Apoptotic nuclei were scored in normal epithelial HEK293 cells or HDF1314 fibroblasts cells and SW480 colon cancer cells treated with a sub-optimal 5-FU concentration of 2.5 µM in combination with 300 nM of MK-8776. At least 200 cells were scored per sample from three different experiments. Proportion of apoptotic nuclei was represented in the bar graph. B, SW480 and HCT116 transfected with siRNA (control or APC) cells were treated with 2.5 µM of 5-FU and 300 nM of MK-8776 and stained with Hoechst to detect apoptosis by nuclear condensation. Proportion of apoptotic nuclei was represented in the bar graph. C, Transient expression of GFP, GFP wild type APC (GFP wt APC) into SW480 cells treated with 2.5 µM for 36 h in combination of 500 nM of MK-8776. At least 100 cells were counted. Positive transfected cells were stained by Hoechst scored to detect nuclear condensation. Proportion of apoptotic nuclei was represented in the bar graph.

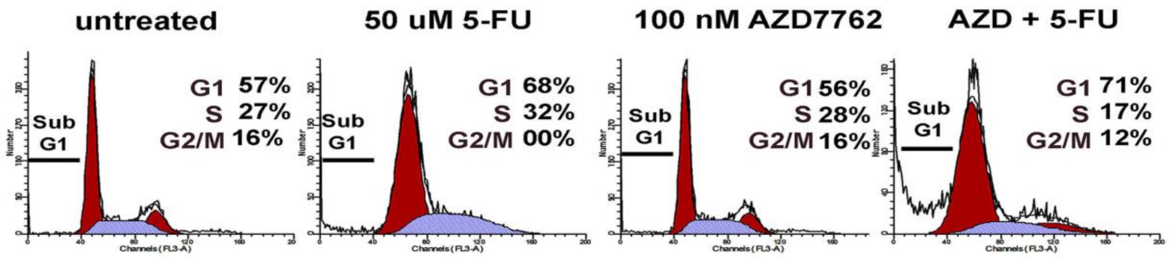

Supplementary Figure S9: Cell cycle profile FACS analysis of SW480 cells treated with 50 µM of 5-FU, 100 nM of AZD7762 or a combination of both drugs.

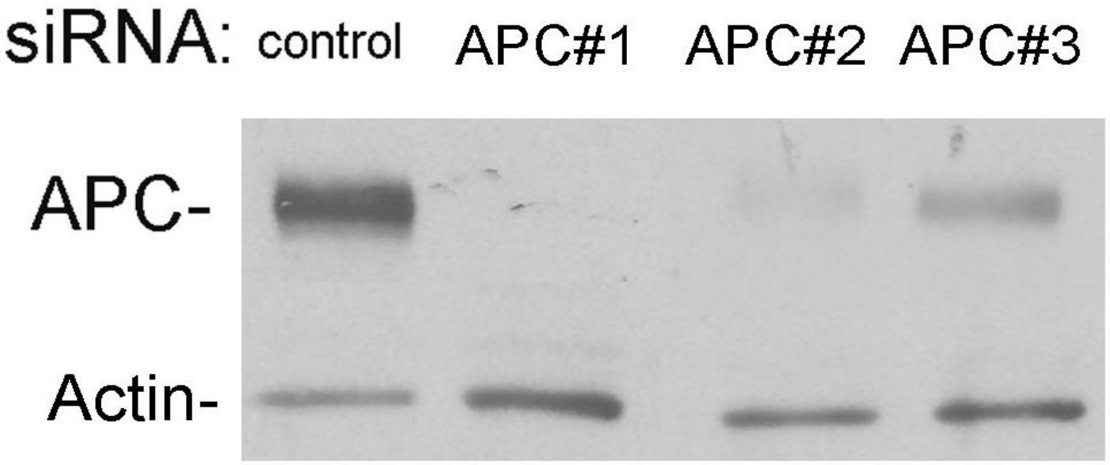

Supplementary Figure S10: Confirmation of APC depletion by siRNAs in SW480 cells treated with 50 µM of 5-FU by Western blot analysis.
